# Can I Do Better Than AI: A Comparative Analysis of a Medical Student Essay Created With and Without Generative AI

**DOI:** 10.7759/cureus.103357

**Published:** 2026-02-10

**Authors:** Nabihah Hussaini, Susan M Astley, Adam Perrett, Elaine F Harkness

**Affiliations:** 1 School of Medical Sciences, The University of Manchester, Manchester, GBR; 2 Division of Informatics, Imaging and Data Sciences, School of Health Sciences, Faculty of Biology, Medicine, and Health, The University of Manchester, Manchester, GBR

**Keywords:** artificial intelligence, chatgpt, large language models, medical education, medical student performance evaluation

## Abstract

Background

With the increasing integration of generative artificial intelligence (AI) tools in education, questions have emerged about their effectiveness in academic tasks such as reviewing literature and writing essays. This paper evaluates whether generative AI could outperform a third-year medical student writing a literature review.

Methods

A third-year medical student wrote a 3000-word literature review on the prevention of breast cancer following University guidelines. Three prompting methods were used to generate essays with the same title and guidance using ‘ChatGPT-4o’ (OpenAI, San Francisco, CA, USA). Each essay was then graded alongside the student’s essay by an AI chatbot using the marking scheme provided. The highest-scoring AI essay and the student’s essay were critically compared and analysed. They were then tested for similarity to pre-existing literature using Turnitin, a widely used plagiarism detector.

Results

The student’s essay comprised 3044 words, written in 452 minutes. In contrast, the highest-marked AI-generated text comprised 3260 words, written in only 15 minutes. However, despite efficiency in time, the AI-generated essay demonstrated reduced originality, receiving a Turnitin similarity score of 46%, compared to the human-written score of 26%, indicating a greater overlap of content from existing sources. Furthermore, it was found that a single prompt was not sufficient to produce a high-quality, original, AI-generated essay; instead, a large series of instructions was required to produce a suitable review.

Conclusions

Generative AI can assist students by complementing their efforts, but it produces work that may lack originality. Further research is required to improve AI’s ability to generate original, in-depth content.

## Introduction

Background

In the rapidly evolving landscape of medical education, the integration of artificial intelligence (AI) tools has sparked both excitement and apprehension. AI’s capability to process vast amounts of data and generate coherent, relevant content presents new opportunities for enhancing learning. However, the question remains: can medical students outperform AI when writing essays? This paper presents a comparative analysis of literature reviews on breast cancer prevention written by a third-year medical student and generated using a large language model (LLM) [1].

LLMs can generate large volumes of text within seconds of being given a singular prompt - the opening sentence of this background section was written in 13 seconds by ChatGPT-4o (OpenAI, San Francisco, CA, USA), a generative language model launched in 2022 [2]. An LLM is an advanced AI system designed to understand and generate human language. Trained on extensive datasets using deep learning methods, they can perform a range of natural language processing tasks, including text generation, summarisation, and translation [3]. ChatGPT is one of the most widely known LLMs, designed to produce coherent, contextually appropriate responses to user input. ChatGPT rapidly gained popularity in fields such as education, content creation, and programming, reaching 100 million users in its first year. Its success led to several major tech companies accelerating the release of their own models - Microsoft’s ‘Copilot’, Google’s ‘Gemini’, and Meta’s ‘Llama’ [2]. These tools are now increasingly integrated into professional and academic settings, raising questions about their capabilities compared to human users.

Unlike earlier AI models, which were primarily designed for limited-domain question answering or classification tasks, ChatGPT exhibits multimodal capabilities. It can respond to exam-style questions, write executable code, and simulate gameplay in text-based environments such as tic-tac-toe. These functionalities reflect the broad utility of advanced LLMs in both academic and applied settings [1,4,5].

ChatGPT-4o has shown medical knowledge equivalent to that of a third-year medical student, having passed all three steps of the United States Medical Licensing (USMLE) exam, scoring between 52.4% and 75% - often regarded as one of the most challenging medical licensing examinations worldwide [6-8]. Much interest has been garnered in just how far generative AI can go - where are the limits, or are there any limits? As AI’s interface improves rapidly, its potential to enhance medical education cannot be ignored. However, it is firstly crucial to assess whether AI can truly replicate or even outperform human intuition, clinical judgement, and scientific knowledge. While AI-generated content may, at first glance, appear coherent and persuasive, some researchers argue that tools like ChatGPT do not engage in genuine reasoning or cognitive processing. For instance, a paper published by the Massachusetts Institute of Technology in 2023 highlights that, although ChatGPT can produce human-like responses, it lacks self-awareness, memory, and understanding, making it fundamentally different from the way human experts reason through complex problems [9].

This study aims to explore one potential use of generative AI in medical education, focusing on ChatGPT-4o’s ability to compile, analyse, and present complex medical literature accurately. It also examines whether ChatGPT-4o can produce original content without evidence of plagiarism.

## Materials and methods

To conduct this research, a comparison will be made between a literature review on breast cancer prevention written by a medical student (NH) and one generated by ChatGPT-4o, the most up-to-date ChatGPT model at the date of the experiment. NH is a third-year MBChB candidate who has previously demonstrated strong academic performance, achieving a distinction in an earlier university literature review assignment.

Choosing a research question

To understand the capabilities of AI, a topic was selected that was unfamiliar to the student but sufficiently challenging, with a broad literature - primary and secondary prevention methods of breast cancer. This is an area of active research: using the three keywords prevention, breast, and cancer, there were approximately 3.5 million results in Google Scholar.

Writing the student essay

The student-generated literature review began with the creation of an essay plan outlining the questions the review would answer. This was produced independently by the student prior to any use of ChatGPT-4o to limit any bias.

The essay plan was subdivided into three sections: (I) Section 1: Introduction and Overview of Breast Cancer - 1.1: Overview and Epidemiology, 1.2: Modifiable Risk Factors, 1.3: Non-modifiable Risk Factors; (II) Section 2: Primary Prevention of Breast Cancer - 2.1: Chemoprevention, 2.2: Secondary Prevention; (III) Section 3: Conclusion and Bibliography.

Research papers were sourced from PubMed and Google Scholar. The full set of search terms employed can be seen in Table 1. Each search term was selected independently by the student. The student cited 20 papers in Section 1, 13 in Section 2, and produced a bibliography using Zotero [10].

**Table 1:** Search terms selected by the student to identify relevant literature for the student-written literature review. Citation numbers in the final column correspond to references included in the review, selected based on relevance, recency, and alignment with the research question.

Source	Search Term	Results	Citation
PubMed	Prevention of breast cancer	63,857	[11]
			[12]
			[13]
	Modifiable and non-modifiable risk factors for breast cancer	1509	[14]
	Family history and risk of breast cancer	6591	[15]
	BRCA in breast cancer	6378	[16]
Google Scholar	Most common cause of cancer deaths in women	1,020,000	[17]
	Modifiable risk factors for breast cancer	232,000	[18]
			[19]
			[20]
			[21]
	Link between risk of breast cancer and age of menopause	187,000	[22]
	Link between breast cancer development and alcohol	1,040,000	[23]
	Link between breast cancer and cigarette smoking	251,000	[24]
	Link between breast cancer and hormone replacement therapy	265,000	[25]
			[26,27]
			[28]
	BMI ranges	1,140,000	[29]
	Physical activity and breast cancer	2,900,000	[30]
	Chemoprevention of breast cancer	195,000	[31]
			[32]
			[33]
			[34]
	The Lancet Breast Cancer Commission	152,000	[35]

Given the large number of results generated by each search term, a systematic approach was used to select relevant sources. Priority was given to peer-reviewed journal articles, particularly systematic reviews and large-scale research studies published in the last 10 years. Articles were included if they directly addressed breast cancer prevention strategies, risk factors, or relevant epidemiological data. Studies with small sample sizes, non-English publication language, or lacking robust methodology were excluded. Additionally, authoritative sources, such as the NHS and WHO guidelines, were included for clinical relevance.

The literature review was written in a structured manner, guided by the University of Manchester MBChB Literature Review Marking Guidelines. The writing process began with an essay plan outlining key sections. Research findings were integrated section by section, ensuring logical flow. The assessment criteria were actively used to refine arguments, ensure depth of analysis, and structure the discussion in alignment with academic standards.

Creating an AI-generated essay

Three approaches were utilised to generate an AI-written literature review using ChatGPT, an LLM popular with medical students.

Method 1

**Figure 1:**
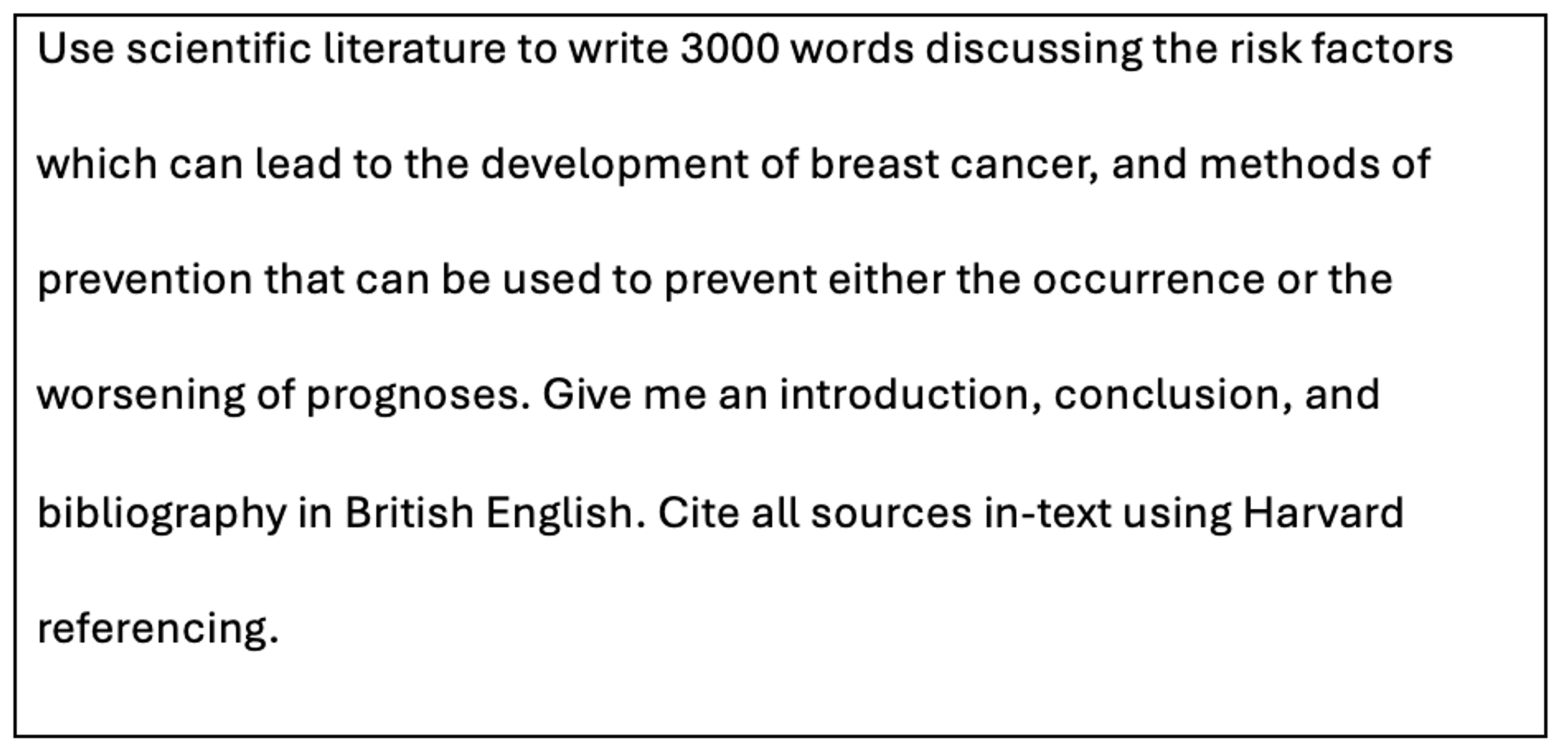
Broad prompt entered into ChatGPT-4o to elicit the ‘lazy student’ response (Method 1).

The first method was one that might be employed by a ‘lazy student’, whereby one broad prompt (Figure 1) was entered into ChatGPT with no further instructions, and the first output generated was accepted as the finished product.

Method 2

The second method, which we refer to as the ‘diligent student’, involved submitting a series of prompts to guide ChatGPT. This method, known as ‘chain-of-thought’ prompting [36], reduces large tasks into smaller steps for improved accuracy and precision. Each paragraph generated was reviewed to tailor subsequent prompts to obtain an improved output.

**Figure 2:**
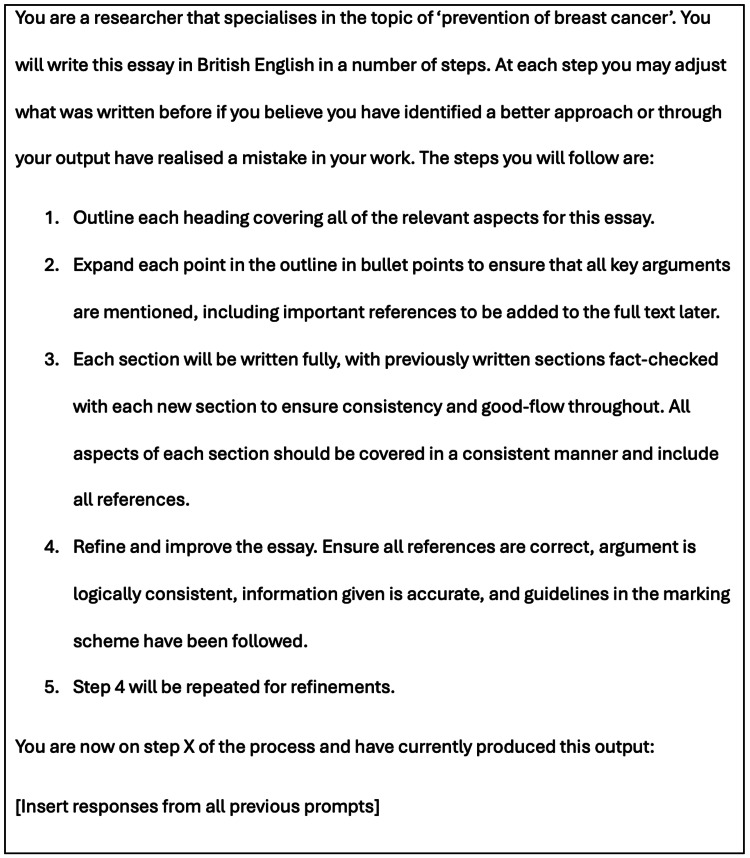
The five-step process entered into ChatGPT-4o to generate the first chain-of-thought literature review (Method 2).

To generate the report, the learning outcomes were outlined, and the University of Manchester MBChB ‘Literature Review Marking Guidelines’ were attached, with instructions to ChatGPT-4o to structure the report accordingly. A series of five steps was provided (Figure 2), and the time taken to generate the responses was recorded. Since previous research has suggested that creating a ‘persona’ when using LLMs can improve responses - especially when this is a field specialist in the topic of interest - the persona of a breast cancer specialist was used for the generation of this essay [37]. 

Each prompt was entered into ChatGPT-4o. The outline was produced and followed by the literature review.

Method 3

**Figure 3:**
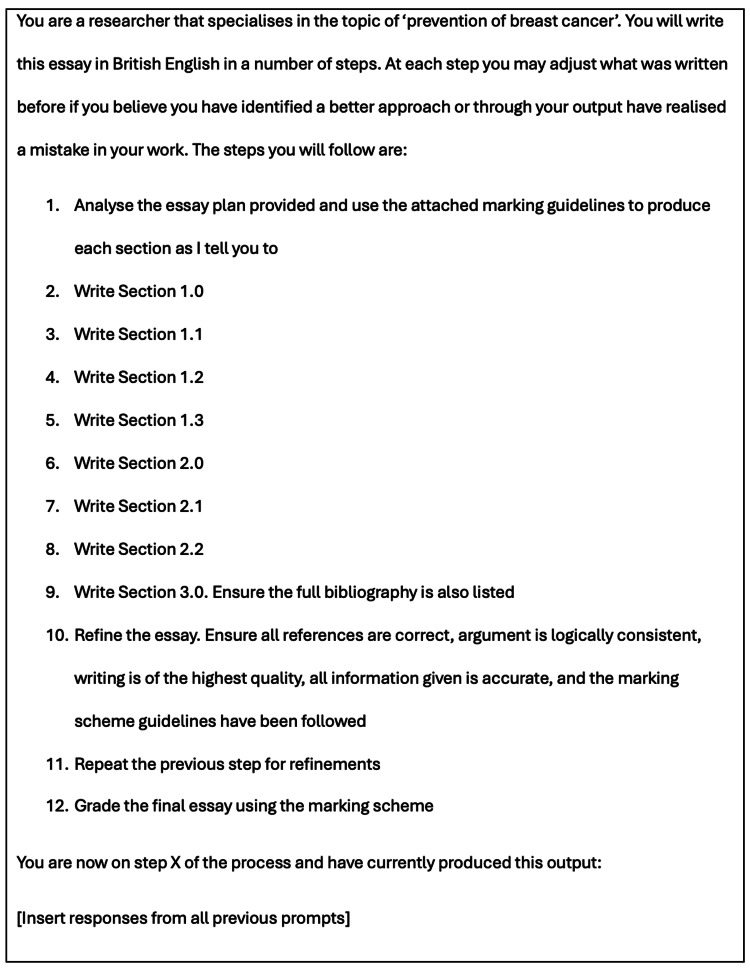
The 12-step process entered into ChatGPT-4o to generate the second chain-of-thought literature review (Method 3).

The third approach used a more refined 12-step chain-of-thought process (Figure 3), but, in addition, ChatGPT was provided with the essay plan used in the production of the student’s essay and an instruction to generate the essay section by section. Each prompt was entered individually to guide ChatGPT through the process. The persona of a breast cancer specialist was again used for the generation of this essay [37]. 

To facilitate comparability, the chatbot was given the same essay plan developed for the student-written review. Each section was generated according to the marking guidelines, but otherwise, the prompts were similar to those used in the second AI-generated essay.

The final version of the literature review was compared with the student-written essay.

Assessment of the essays

To assess essays, three objective metrics were used: time taken to write, which was evaluated using an online stopwatch; University of Manchester Literature Review Marking Guidelines; and a plagiarism detection tool, Turnitin [38]. Assessment against the University guidelines was undertaken by ChatGPT-4o, with domains covering content, use of literature, organisation, and presentation. ChatGPT-4o was required to assign a percentage mark based on the assessment criteria (<50% unsatisfactory, 50%-69% satisfactory, 70%-79% honours, and >80% distinction).

Each literature review was passed through Turnitin, which compares submitted text with published and unpublished work to generate a similarity score, indicating the overlap between submitted work and pre-existing content; a high score raises concerns about potential plagiarism. Turnitin does not provide definitive guidance on what similarity score should be considered high, instead leaving it to the interpretation of the assessor, who must exercise judgment in relation to contextual factors, such as the nature of the assignment.

Finally, each essay was also subjectively evaluated by the student, the findings of which can be seen in the Results section.

## Results

The student-written review spanned 3,044 words across 27 paragraphs and cited 25 studies - 23 were in peer-reviewed journals, and two references were to health organisation websites, with 11 of these 25 sources published in the last 10 years. The student-written essay required 452 minutes (7.5 hours) of research and writing time.

ChatGPT-generated literature reviews varied by prompt complexity: the ‘lazy student’ approach (Method 1) produced 740 words in under one minute; the five-step chain-of-thought process (Method 2) yielded 1,117 words in three minutes; and the 12-step process (Method 3) generated 3,260 words, citing 42 studies in just 15 minutes. The student’s effort thus took 30 times longer than the most detailed AI-generated counterpart, as summarised in Table 2.

**Table 2:** Key characteristics of student-written and AI-generated literature reviews (Methods 1-3), including total word count, time taken to write (minutes), number of paragraphs, and number of references cited.

Essay Type	Word Count	Writing Time (Minutes)	Number of Paragraphs	Number of References
Student-Written	3044	452	27	25
Method 1: Single Prompt AI	740	<1	6	8
Method 2: 5-Step AI	1117	3	10	14
Method 3: 12-Step AI	3260	15	28	42

Subjective comparison of the student-written and 12-step AI-generated essays

**Figure 4:**
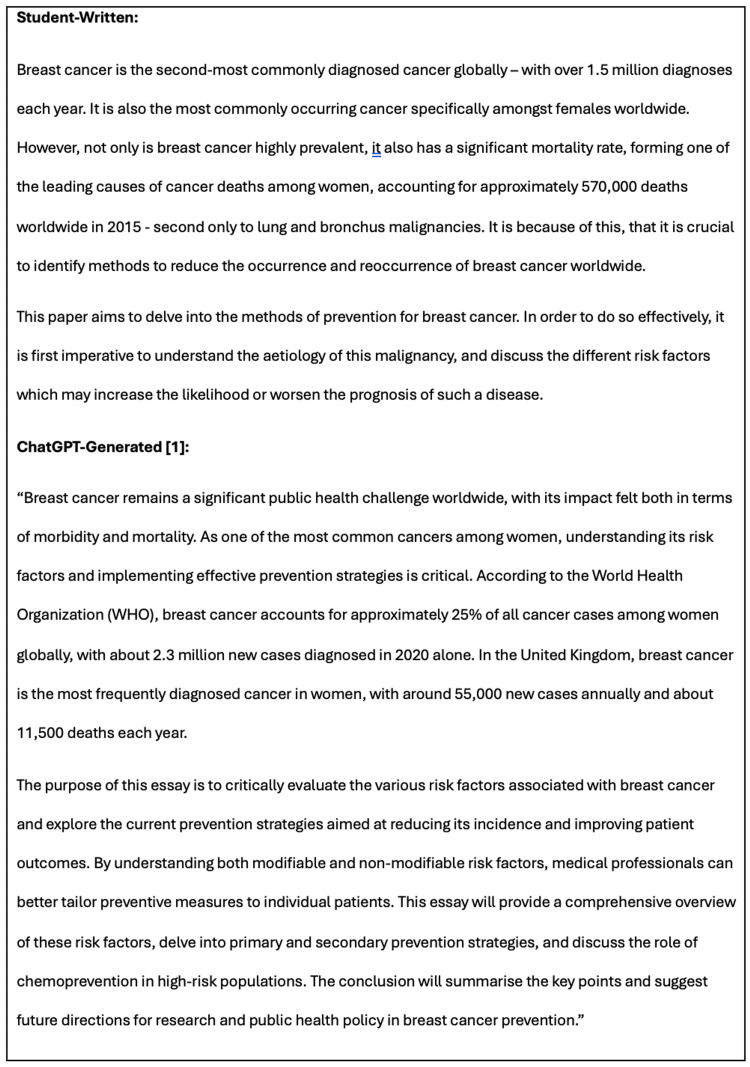
Introduction for both the student-written and AI-generated (Method 3) literature reviews.

Both essays (student-written and the 12-step ChatGPT version) demonstrated the importance of breast cancer epidemiology. A similar structure was used, outlining key statistics, discussing the aim of the paper - to further understand the aetiology of breast cancer - and ascertaining preventative methods. Figure 4 illustrates the ‘Section 1: Introduction and Overview of Breast Cancer’ of both literature reviews.

**Figure 5:**
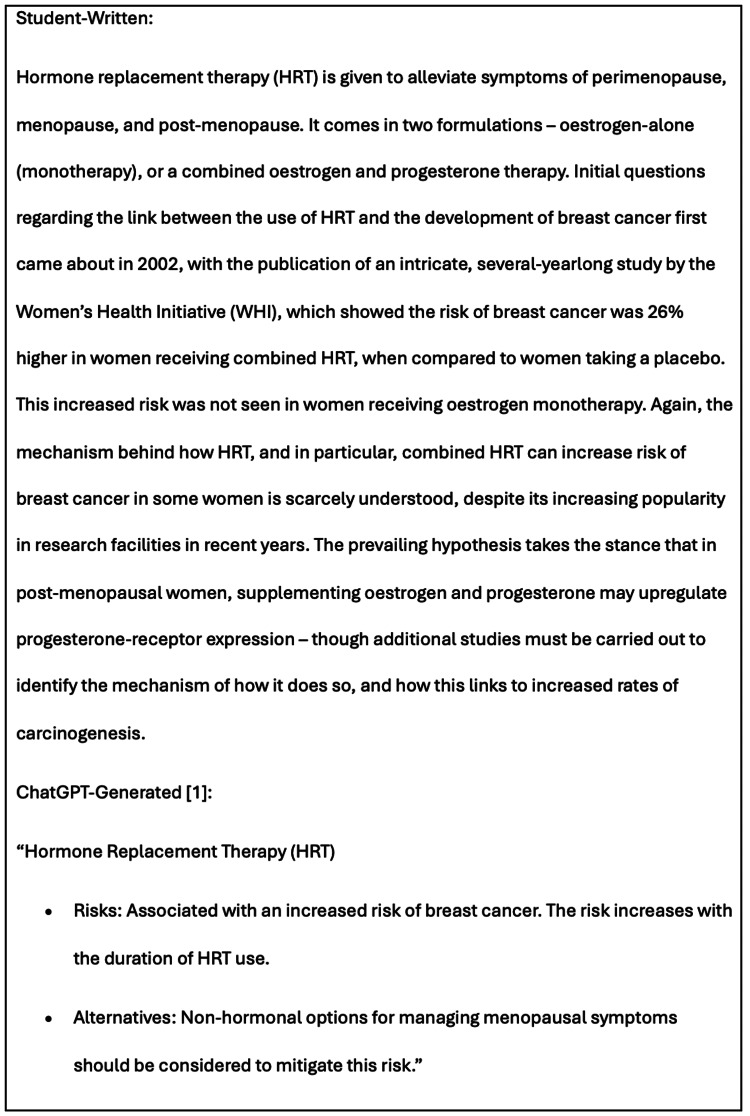
Partial corresponding sections from Section 1.2, ‘Modifiable Risk Factors’, in both literature reviews - the student-written and the 12-step AI-generated (Method 3) - illustrating the lack of depth in the ChatGPT-generated essay.

When discussing risk factors, both essays categorised these into modifiable and non-modifiable. However, ChatGPT’s explanations were brief and lacked depth when compared to the student-written analysis, which offered a more extensive discussion on complex pathophysiology, as seen in Figure 5, which displays corresponding snippets of ‘Section 1.2 - Modifiable Risk Factors’ of both literature reviews.

**Figure 6:**
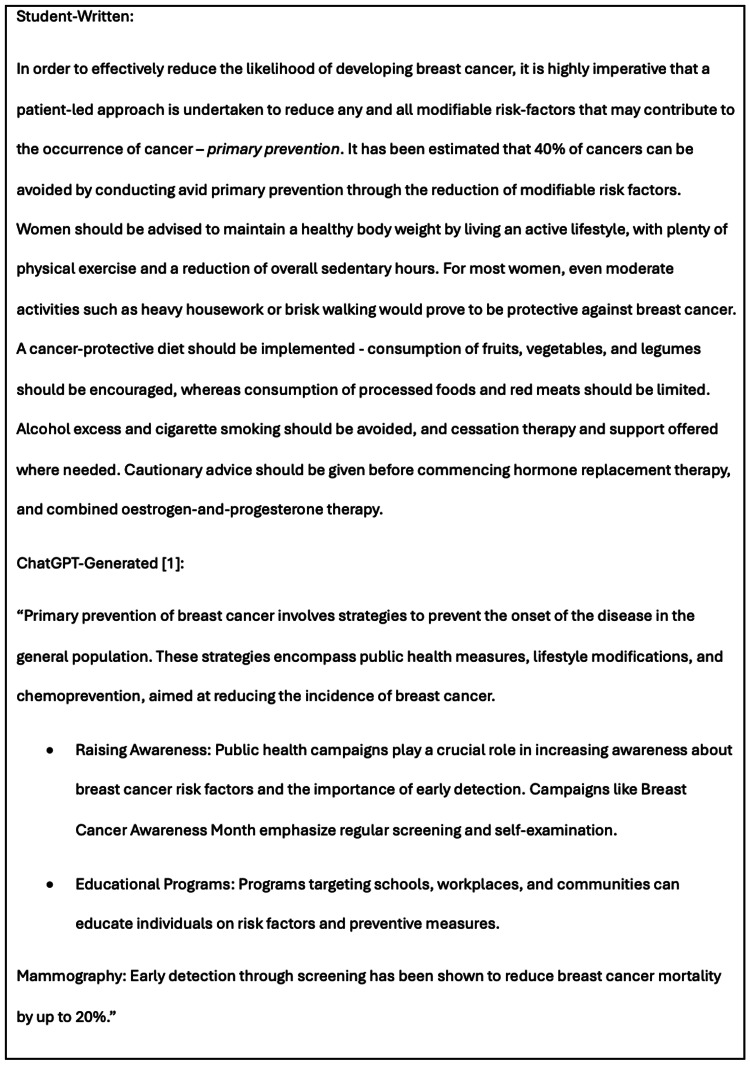
Partial corresponding texts from Section 2, ‘Primary Prevention of Breast Cancer’, in the student-written and 12-step AI-generated (Method 3) literature reviews, highlighting differences in the content discussed by the two authors.

In ‘Section 2 - Primary Prevention of Breast Cancer’, both essays categorised approaches to prevention as either primary or secondary, though the emphasis differed. The student-written essay emphasised targeting modifiable risk factors, whereas ChatGPT stressed public health awareness campaigns (Figure 6).

Both reviews discussed techniques for secondary prevention. Whilst the student-written essay outlined in significant detail the mechanism of action of the main class of chemoprevention - Selective Oestrogen Receptor Modulators (SERMs) - the report written by ChatGPT-4o offered a wider range of information on alternative forms of chemoprevention, expanding breadth but lacking detail.

**Figure 7:**
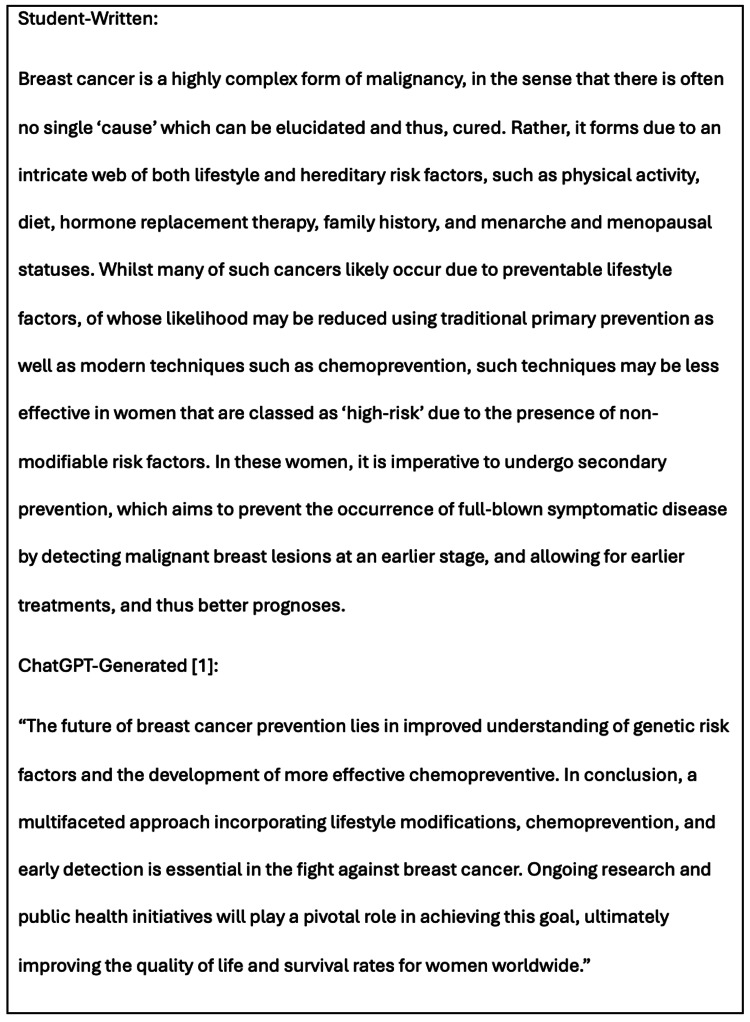
Section from the concluding statements of both the student-written and 12-step AI-generated (Method 3) literature reviews.

Both literature reviews conclude by summarising the key findings and emphasising a multifaceted approach to breast cancer prevention, as shown in Figure 7. The ChatGPT essay begins with a grammatically incorrect sentence.

Assigning a grade using ChatGPT-4o

To objectively grade the essays, marking guidelines from the University of Manchester were uploaded into ChatGPT alongside each report. These guidelines assess both content quality and the technical quality of writing, with emphasis placed on content, addressing the topic, relevance of information, use of literature, citations, and overall presentation.

The 12-step chain-of-thought AI-generated literature review scored a total of 85% - a distinction. Maximum points were achieved for ‘relevance of information’ and ‘addressing the topic’, with points deducted for inconsistencies in referencing and language.

The student-written report also received a distinction, with a mark of 87%. While receiving fewer points for content and relevance, the student's essay compensated in presentation and language.

A summary of the results by section can be seen in Table 3.

**Table 3:** Summary of results from the report cards of the AI-generated (Method 3) review and the medical student-written review, according to the University of Manchester Literature Review Marking Scheme.

	ChatGPT-4o	Medical Student
Content		
Addressing the topic	10	9
Relevance of information to the topic	10	9
Interpretation and accuracy of information	9	8
Subtotal	29/30	26/30
Use of Literature		
Range of literature cited	9	9
Citation of literature	8	8
Subtotal	17/20	17/20
Organisation		
Subdivision of report	10	9
Use of text, tables, and figures	8	8
Subtotal	18/20	17/20
Presentation		
Use of English	8	9
Presentation	5	9
Subtotal	13/20	18/20
Total	77/90 (85%)	79/90 (87%)

Similarity with other sources

The student-written review received a similarity score of 26% in Turnitin, of which 23% comprised matches from the internet, with 16% from publications in the literature, and 5% from other student works.

The ChatGPT-generated review received a similarity score of 46%-20% higher than the student-written essay, of which 38% of the content was matched to sources from the internet, 29% from publications, and 26% from previous student papers. 

## Discussion

The objective of this study was to establish whether generative AI could generate essays of a similar standard to the work of medical students.

This work found that, using detailed prompts, ChatGPT was able to generate a high-quality 3,260-word literature review in 15 minutes, whereas it took a third-year medical student approximately 452 minutes to write a 3,044-word review of a similar standard on the same topic.

ChatGPT-4o also performed well in its use of citations. While LLMs are infamous for ‘hallucinations’, where information is fabricated, all 42 references were verified as correct [39]. However, on subjective analysis, parts of the review lacked depth, providing only limited discussion of areas involving complex scientific knowledge.

When graded using the university guidelines intended for a piece of work undertaken by second-year MBChB students, the ChatGPT essay received a score of 85%. Whilst this falls into the highest-grade boundary, the report still fell short of expectations, as the LLM was asked to produce the essay according to the criteria by which it subsequently scored itself. Despite having knowledge of the specifications needed to obtain a perfect score, it failed to do so.

Additionally, ChatGPT showed difficulty generating large texts from single prompts, as seen in the first two generated essays. Only the 12-step essay reached the goal of 3,000 words, requiring individual section prompts. The need to break down tasks into step-by-step instructions diminishes AI’s key advantage of speed and efficiency, with more time required to create detailed guidance for the LLM than was initially anticipated. Additionally, ChatGPT displayed a limited memory - it is said to maintain memory of up to 10,000 words, though even within this, it often lost the context of its instructions, particularly with multiple prompts.

Language inconsistencies were noted, with ChatGPT frequently defaulting to American English despite explicit requests for British English.

Finally, the Turnitin score of 46% highlights a major concern for originality. The high similarity score of the ChatGPT-generated review raises concerns in the context of academic integrity. Many institutions set threshold levels to flag potential plagiarism or over-reliance on source material, and a score approaching 50% would likely prompt further scrutiny. The AI-generated review scored 20% higher than the student-written text, indicating issues with over-reliance on phrases and sentences from pre-existing work.

Similar findings have been observed in other academic disciplines. A 2024 study comparing ChatGPT-generated essays with those written by university students in the humanities reported that AI-produced essays often achieved high marks but, nevertheless, lacked the depth, critical analysis, and nuanced argumentation demonstrated by human writers [40]. This aligns with the present study, in which ChatGPT produced a well-structured, high-scoring review but showed limitations in deeper scientific reasoning and interpretation. ChatGPT versus human essayists also highlighted that AI tends to excel in coherence and surface-level organisation, while struggling with originality and sophisticated critical engagement - patterns mirrored in our findings.

The main limitation of the student-written report was the time-consuming, labour-intensive process. There is also a higher risk of selection bias inherent in human writing; an example of this was the omission of information about aromatase inhibitor drugs for chemoprevention, which ChatGPT included.

The high score achieved by ChatGPT highlights its potential for medical education and the provision of public information. The key lies in the art of collaboration - using AI as a tool rather than a replacement. LLMs may assist with initial searches or overviews, allowing time for in-depth human analysis.

However, the limitations of AI cannot be ignored. One must consider the ethical concerns - issues of plagiarism and reliance on pre-existing content, and the need for transparency with AI-generated content. 

Strengths and limitations

Strengths of this study include the direct, controlled comparison between the AI- and student-generated literature reviews on the same topic, using identical marking criteria. This allowed for a fair assessment of quality, time efficiency, and originality. The use of multiple AI prompting strategies also provided insights into how different prompting techniques impact AI performance.

However, the study has a number of limitations. Only one student-written essay and one topic were used, which may limit generalisability. Secondly, the assessment of each essay was partially conducted by the same AI system being evaluated, and by the student who wrote the initial essay, which introduces the potential for bias. Future studies can limit this by implementing an independent assessment by a neutral, third-party assessor.

## Conclusions

This comparative analysis between ChatGPT-generated and student-written literature reviews on the prevention of breast cancer revealed insights into the capabilities and limitations of each approach. The results suggest potential for the use of LLMs in medical education by enabling students to research topics in a time-effective way. However, this efficiency may come at the expense of originality. The results also demonstrate the need for evolving assessments so that students are tested on their knowledge and understanding, rather than on their ability to format work. There are continuing concerns about the accuracy and verification of LLM-generated text in the form of hallucinations, which is a skill that medical students of the future will need to develop. The position of publication of work written with the assistance of generative AI is also rapidly evolving, with some types of research more vulnerable to a lack of originality than others.

To fully harness the potential of generative AI, further research is required to improve AI’s ability to generate original content and avoid misinformation. The integration of generative AI in medical education should focus on using its strengths to enhance students’ work, rather than replacing the process completely.
